# Induction of spermatogenesis in men with hypogonadotropic hypogonadism

**DOI:** 10.1007/s10815-020-02058-0

**Published:** 2021-01-11

**Authors:** Guy C. Morris, Esther Lloyd-Evans, David J. Cahill

**Affiliations:** 1 Reproductive Medicine Unit, University College London Hospitals, Fitzrovia, London, NW1 2BU UK; 2 St Michael’s Hospital, Southwell St, Bristol, BS2 8EG UK; 3 St Michael’s Hospital, University of Bristol, Southwell St, Bristol, BS2 8EG UK

**Keywords:** Spermatogenesis, Gonadotropins, Cost-effectiveness, Pregnancy, Sperm

## Abstract

**Purpose:**

We compared our clinical experience to international standards, assessed by response to treatment and pregnancy rates to ensure our results were comparable.

**Methods:**

Men presenting with azoospermia related to hypogonadism were recruited into a treatment programme which was managed by one person over 8 years in a secondary care facility. Treatment followed published management plans using urinary gonadotropins. Data were collected on success rates in spermatogenesis, as well as variables which might predict success, and costs. Statistical analysis used non-parametric methods.

**Results:**

Of 16 men with HH, 14 achieved spermatogenesis, and 9 had sperm cryopreserved. Of those 14, 6 were successful in achieving a pregnancy with their partner from assisted conception (including ICSI) and one after natural conception. Factors identified to identify men likely to be successful in treatment were whether testicular volume was larger at onset of gonadotropins (median 10 mL) with a trend towards greater success if the cause developed after puberty. Mean treatment costs per man treated amounted to GP£4379/UD$5377 (figures for September 2020).

**Summary:**

Success rates from this treatment should exceed 70% in most clinical settings. The likelihood of success improves when testicular volume exceeded 10 mL at initiation of treatment and a trend exists whereby success is more likely whereby when hypogonadism developed after puberty. Treatment costs are at a level likely to benefit quality of life, supporting the delivery of this treatment and where necessary and possible, funding it in line with other fertility treatments. This treatment should be available much more widely as a management option for men with hypogonadism, allowing them to father a biological child, rather than using donor sperm.

## Introduction

Disorders of sperm function and production are found in 36% of the infertile population [1], but related endocrine causes are rare. One large cohort study of men with sperm disorders found that only 18 of 1035 men (1.7%) had a “clinically significant” endocrine disorder [2]. Severe forms of endocrine-related sperm production disorders include those dysfunctions of the hypothalamic-pituitary axis which result in hypogonadotropic hypogonadism, either are related to anosmia (Kallmann syndrome) or are idiopathic (IHH). Hypogonadotropic hypogonadism is rare, occurring in fewer than one man in 500 [1]. In the paper referred to above, hypogonadotropic hypogonadism was the cause of the man’s infertility in only 2 of the 1035 (0.3%) [2]. This condition is therefore rare but amenable to long established, effective treatment, by regular gonadotropin injections, usually self-administered [3]. But what is the cost of this treatment and … do some clinics shy away from treating men with IHH because of cost?

Response to treatment is related to the underlying cause of HH: men with post pubertal acquired HH have the best response to treatment and achieve higher pregnancy rates [4]. Combined (hCG and FSH) gonadotropin use treats both hypothalamic and primary pituitary causes of HH and may require quite prolonged stimulation, up to 24 months [5]. Other predictors of a positive response to treatment include larger baseline testicular size and absence of undescended testes. Negative predictors for response to treatment include smaller testicular volume and pubertal status [6].

Our clinical work is undertaken in a secondary care hospital, not in a specialist unit, and we wanted firstly to ensure our results were comparable with national and international standards, secondly to consider if there were any predictive factors evident between the successful and unsuccessful groups and finally to estimate a cost for the treatment. Published literature suggests that 60–75% of men should have sperm (> 1.5 × 10^6^ sperm/mL) on completion of treatment—that is, after 6 months [5, 7]. The aim of treatment in our unit is to induce sufficient numbers of sperm so as to allow sperm to be stored and used at a later date, with the expectation that would be using in vitro fertilisation associated with intracytoplasmic sperm injection.

## Methods

We undertook an evaluation of our spermatogenesis outpatient service, with data collected prospectively from January 2008 to August 2017. Treatment regimens were unchanged over that time and all treatments were managed by one of us (DJC). Data were collected on all men undergoing gonadotropin therapy, and those data were corroborated with data from a local licenced sperm freezing service. As this was a service evaluation only, designed to ensure published standards were being met, ethical approval and informed consent were not obtained.

We used a treatment approach in line with published effective protocols [5, 7]. These involve hCG injections (2000 IU IM) three times a week followed by serum testosterone measured at 4 weeks. Once serum testosterone exceeded 10 nmol/L (288 ng/dL) in line with other published studies [5, 7], follicle-stimulating hormone was added (FSH 75 IU SC) three times a week. Different proprietary formulations of hCG and FSH injections were used throughout the span of the review—generally the least expensive that the hospital pharmacy could purchase, and generally a urinary derived gonadotropin product. Seminal fluid analysis was performed 4 months after commencing FSH treatment. If there were no sperm present, the dose of FSH was increased to 150 IU SC three times a week, and a further sample assessed 4 months later. Once sperm were observed in the sample, arrangements were made for sperm to be freeze stored. If sperm were not seen on the second sample, gonadotropin therapy was discontinued.

Data were collected on the underlying cause for HH, initial testicular volume (using a Prader orchidometer), initial gonadotropin levels, pre-treatment testosterone levels, initial semen analysis values, starting hCG doses and dose changes, post hCG testosterone levels, FSH doses and changes, post FSH testosterone, post FSH semen analysis, spermatogenesis outcome, sperm storage success, conception rate, duration from initiation of treatment to spermatogenesis, costs of treatment (the pharmacy costs of the gonadotropins only, not investigations, assessments or the costs of freezing) and conception. For men who produced sperm and those who did not, these variables were compared using a non-parametric method (Mann-Whitney *U* test). Statistical significance was considered when *p* values less than 0.05 were present. The data on gonadotropin costs were provided by the hospital pharmacy for each individual patient.

Of the 16 men who presented for treatment, 14 had female partners, and 2 were referred directly without partners. No data on female partners was collected on those two men. All these men on referral were taking androgen replacements prescribed by their primary or secondary care physicians. On referral, and before beginning treatment, androgen replacements were stopped for 6 weeks, in line with other publications [5, 7]. The local health service commissioners ruled that couples could not be referred if the man were over 55 or the woman over 40. This service review focussed on the outcome of spermatogenesis and we collected limited data on the female partners—these included age and serum FSH as these are the primary determinants of ovarian function and reserve for women [5] (other reliable measures were not in use through the time span of the review). The age provided in the data are derived from the ages at first referral. Not all women had FSH levels measured, as the primary focus was on spermatogenesis in the male.

## Results

During the time studied, 16 men with HH underwent induction of spermatogenesis with gonadotropins. Questioning did not reveal any use or abuse of recreational androgen supplements in these men. Table 1 provides the individual details by age and diagnosis of the men and their partners (together with details of whether spermatogenesis was effective, resultant treatments and any pregnancies and costs). Of the 16 men, 14 were azoospermic at the start of treatment, 14 achieved spermatogenesis (88%) and 11 had sperm frozen (68% of the 16; 78% of the 14).

**Table 1:** Individual details by age and diagnosis (with details of whether spermatogenesis was effective, resultant treatments and pregnancies, and costs)

Number	Maleage (years)	Malediagnosis	Femaleage (years)	First femaleFSH (IU/L)	Initial testicularvolume(mL)	Initial FSH (IU/L)	Success (sperm)achieved	Days oftreatment	Sperm stored, conception attempted/achieved by what means, pregnancies		Cost of treatment (GBP)
1	*32*	1	33	ND	*2*	< 0.5	No	571	*None stored*		*10,519.42*
2	*34*	2	27	5.2	*8*	1.4	Yes	b	*Stored, IVF*^*e*^*/ICSI* ^*f*^*× 2*	None	
3	*50*	3	34	5.5	*12*	< 0.5	Yes	c	*None stored*		
4	*29*	1	29	ND	*2*	0.8	Yes	c	*Stored, IVF*^*e*^*/ICSI* ^*f*^	Biochem^d^	
5	*35*	3	30	8	*10*	6.5	Yes	602	*Stored, IVF*^*e*^*/ICSI* ^*f*^*× 2*	Twins, none	*3945.60*
6	*44*	3	32	7	*12*	8.9	Yes	623	*None stored*		*5964.48*
7	*50*	4	40	5.9	*15*	< 0.5	Yes	434	*Stored, IVF*^*e*^*/ICSI* ^*f*^*× 3*	One baby	*1747.80*
8	*34*	4	33	ND	*8*	0.9	Yes	119	*Stored, IVF*^*e*^*/ICSI* ^*f*^*× 3*	One baby	*1617.84*
9	*38*	4	a	ND	b	1.9	Yes	c	*Stored, no treatment*		
10	*50*	4	33	ND	*10*	7	Yes	c	*Stored, IVF*^*e*^*/ICSI* ^*f*^*× 1*	None	
11	*28*	2	a	ND	*2*	< 0.5	No	90	*None stored*		*136.08*
12	*35*	1	34	ND	b		Yes	c	*Stored, no treatment*		
13	*38*	1	31	ND	b	< 0.5	Yes	125	*Stored, no treatment*		*583.92*
14	*39*	2	35	5.8	b	< 0.5	Yes	455	*None stored*		*6008.88*
15	*32*	2	25	4.1	*2*	< 0.5	No	936	*Stored, IVF*^*e*^*/ICSI* ^*f*^*× 1*	One baby	*10,958.40*
16	*31*	1	34	ND	*5*	< 0.5	YES	571	*Natural conception stored, IVF*^*e*^*/ICSI* ^*f*^*× 1*	One, one	*3946*

Male diagnosis: 1: idiopathic hypogonadotropic hypogonadism (and Kallmann syndrome); 2: pituitary failure as a result of hypothalamic disease, medical or surgical in origin; 3: hypogonadotropic hypogonadism secondary to mast cell activation, radiotherapy, or surgery; 4: pituitary failure following on treatment of a hormonally active or inactive pituitary adenoma

*ND* not done

^a^Had no female partner

^b^Testicular volumes not recorded, for those recorded, volumes were concordant on the right and left sides

^c^These men disappeared from follow-up, the number of days of treatment and therefore costs were not established

^d^A biochemical pregnancy with +ve hCG but no sac or FH seen on ultrasound

^e^IVF

^f^ICSI

Median parameters for the successful and unsuccessful cycles of gonadotropin stimulation of spermatogenesis were analysed for all these outcomes: initial testicular volume (mL), initial LH (IU/L), initial FSH (IU/L), initial semen volume (mL), initial testosterone (nmol/L), initial total sperm count (million per ejaculate), first testosterone after 4 weeks hCG, change in testosterone post FSH (nmol/L), change in semen volume post FSH (mL), change in total sperm count following FSH (million per ejaculate), duration to spermatogenesis/treatment cessation (days) and cost of treatment (GB£). There was no difference between the successful and unsuccessful cycles in any parameter except for the initial testicular volume: 10 ± 3.3 mL vs. 2 ± 0.0 mL, respectively, *p* < 0.03. Those who achieved spermatogenesis were more likely to have lower testosterone (but not significantly so, 5.05 ± 14.2 nmol/L vs 12.7 ± 7.6 nmol/L, respectively). One man (patient 15 in Table 1) required additional growth hormone treatment because of his panhypopituitarism, leading to a prolonged period of gonadotropin therapy (a recognised but rare complication in this clinical situation [8]).

Of the 11 men for whom sperm were frozen, 8 proceeded to at least one cycle of assisted conception (using intra-cytoplasmic sperm injection) and 5 achieved a clinical pregnancy from IVF (giving a life birth rate from spermatogenesis of 6 out of 16 (37.5%)—one man conceived naturally with his partner on gonadotropin treatment and had sperm frozen that was later used for assisted reproductive treatment leading to a successful pregnancy). One man and his partner had two cycles of IVF/ICSI—achieving a pregnancy in the first but not the second cycle; two further men and their partners had three cycles of IVF/ICSI—achieving one pregnancy each, respectively. In total, the eight cycles of IVF/ICSI resulted in five pregnancies to date (63% of 8). Three of the men who achieved spermatogenesis with motile sperm did not proceed to having sperm frozen (insufficient numbers in two; decided not to in one case).

Two men did not produce sperm; one had previously been treated for craniopharyngioma prepubertally and one suffered from Kallmann syndrome. None of these underwent any form of surgical sperm recovery. Sperm production in men with acquired hypogonadotropic hypogonadism (11 of 12 produced sperm) appeared to be more successful than with pre-pubertal hypogonadotropic hypogonadism (3 of 4 produced sperm) (though not significantly so).

Of those men who were successful in spermatogenesis, the longest recorded duration of therapy was over 900 days (median 434 days); of those unsuccessful, the longest recorded duration of therapy was 571 days (median 455 days). Costs of treatment were evaluated by drug costs only (not including medical or nursing time) and total costs for all those treated (for the nine men with complete data) were £39411, £4379 per man treated. Costs in Table 1 vary considerably depending on the duration of time hCG alone or hCG and FSH were given. These men have or will all require further treatment with in vitro fertilisation combined with intracytoplasmic sperm injection which will add to the costs—the likelihood of natural conception as happened in one case is small and the time window for natural conception is limited to a few months of normal spermatogenesis. This has been a key element to our counselling of these men before they start treatment. For all the women who had assessment of ovarian reserve, all FSH levels were less than 9 IU/L.

## Discussion

The data in this paper are small and might be dismissed as a result, this being seen as a weakness. However, they are data from one clinical service in a teaching hospital, not from a specialist fertility centre and not from a research programme, offering gonadotropin therapy for spermatogenesis for over 15 years. This review of the outcomes from our clinical service affirms the importance and effectiveness of spermatogenesis as a treatment and supports the provision of gonadotropin induction of spermatogenesis in a secondary care setting and shows that these patients do not have to be managed in a tertiary fertility unit.

Spermatogenesis was achieved in 14 of 16 men (88%); this compares well with published data from in which rates of 60–75% were achieved with gonadotropin therapy [5, 7]. Larger initial testicular volumes were more likely to lead to success at spermatogenesis, as others have found [6]. We found a trend whereby successful treatment was more likely in men with post-pubertal hypogonadism causes, while others found no difference in the cause of the hypogonadism. Others did find that better responses were found in post-pubertal hypogonadism. Our findings are in line with others, all men in our cohort had both hCG and FSH, and all had been previously been on some form of testosterone replacement therapy. All our men administered their alternate day injections; none reported any problems with this or any complications or side effects.

A PubMed search using the terms “cost” and “induction of spermatogenesis” found only one other study examining cost—in that paper, this was within the context of reducing cost of spermatogenesis [7]. Data on cost effectiveness are therefore scanty. Our data suggest that an average drug cost for an individual man is £4379 (EUR4890, USD$5377) (September 2020 exchange rates). For these men who achieve sperm production, median total sperm numbers of 15.9 million will preclude any treatment apart from intra-cytoplasmic sperm injection which will obviously add to the treatment costs. Nonetheless, if adequate numbers can be frozen, those sperms can give rise to further pregnancies in the future. Different gonadotropins were used throughout the review period, and in contract to other studies [7], for much shorter periods of time. Despite that shorter period of spermatogenesis, comparable results were found. For the women, ages were all within the normal reproductive age, and the FSH levels when measured were all within a range considered to be likely to give rise to a normal ovarian response when stimulated by gonadotropins for IVF [9].

Men with HH want to be fertile (almost 60% expressed this in the meta-analysis by Rastrelli et al. [6]). HH may be reversible and fertility can be restored, though not permanently, and this does depend on the underlying diagnosis and whether the onset was pre or post pubertal [4]. Our data and other publications agree that post-pubertal hypogonadotropic hypogonadism is the more amenable to treatment. This is likely to be related to a better germ cell and Sertoli cell proliferation and maturation [10]. In our data, 14 men achieved spermatogenesis—the majority did not achieve normal levels for their sperm parameters—only 1 man did so. Overall, a clinical pregnancy was achieved in five couples in which the men had successful treatment, one by natural conception and the rest using ART, all requiring ICSI in addition. No successful pregnancies were achieved in the group of men that had Kallmann syndrome or pre-pubertal cranial radiotherapy. This contrasts with others who reported a 40% pregnancy rate for Kallmann syndrome [4] but also found that Kallmann syndrome was associated with the poorest clinical outcomes after attempted spermatogenesis [4].

Our success rates compare well with other published data. There are very little published data beyond our own results with which to counsel couples considering treatment in a non-specialist unit providing gonadotropin therapy. We suggest that there is a need to collect these data nationally to inform couples about the likely success of treatment. Costs per man treated to achieve spermatogenesis are low and well within WHO guideline limits for cost-effective treatments [11]. Median treatment duration whether successful or not was of the order of 18–24 months (434 and 455 days, respectively). This is clearly a long time to maintain compliance over, but our experience is that patients seem very dedicated to and complaint with the treatment. Despite this, many clinics (in a UK-based survey) view cost as a major barrier to initiation or continuation of treatment [12].
